# Mild cooling of the feet does not aid night-time vigilance

**DOI:** 10.1186/2046-7648-4-S1-A114

**Published:** 2015-09-14

**Authors:** Ryan Sixtus, Barbara C Galland, James D Cotter

**Affiliations:** 1 School of Physical Education, Sport and Exercise Sciences, University of Otago, Dunedin, New Zealand; 2 Dunedin School of Medicine, University of Otago, Dunedin, New Zealand

## Introduction

Vigilance is related to core temperature (T_C_) and skin temperature (T_sk_). Biological day reflects a high T_C_, alertness and modest T_sk_; night reflects *vice versa *[1,2]. At rest, T_C _is regulated largely via controlling blood flow in extremities (and thus T_sk_); vasodilation strongly predicts reduced vigilance [3] and faster sleep onset[4]. In narcolepsy, high daytime extremity temperatures and a smaller distal-to-proximal gradient (DPG) indicates higher sleep propensity [5]. Cool extremities have been linked observationally to delayed sleep onset in the elderly, and experimentally shown to reduce sleep propensity in narcolepsy [6]. Therefore, the aim of this study was to test the hypothesis that cooling the feet would maintain vigilance during extended wakefulness in healthy adults.

## Methods

A randomised cross-over experiment was completed using nine healthy young adult participants with normal sleep patterns. After providing informed consent, and a daytime familiarisation, they undertook three 3-h laboratory sessions in which water-perfused booties were used to provide Mild cooling, Moderate cooling or no cooling (Control). Sessions were in a dimly-lit room, beginning at 2230. Each 30 min consisted of quiet rest interspersed with a 10-min psychomotor vigilance task (PVT), 7-min Karolinska Drowsiness Test (KDT), and ratings of sleepiness, perceived body temperature and thermal discomfort. EEG spectral powers (theta, alpha and beta) were determined within the PVT and KDT. Analyses were by repeated measures ANOVA (α = 0.05) with post-hoc contrasts with Bonferroni correction.

## Results

Foot temperatures in Control and Mild and Moderate cooling averaged 34.5 (0.5), 30.8 (0.2) and 26.4 (0.1) °C (all *P*<0.01). Yet, the upper-limb DPG remained stable (at ~0.3 °C) regardless of condition (*P *= 0.57). The decline in T_C _(~0.35 °C) was also unaffected by condition (*P *= 0.84), as was vigilance (interaction for response speed: *P *= 0.45). A small and transient reduction in sleepiness was evident with cooling (*P *= 0.046); otherwise sleepiness and vigilance deteriorated in conjunction with the fall in T_C _in each condition (*r *> 0.80). Participants felt cooler throughout both cooling trials, but thermal comfort was unaffected (*P *= 0.43), as were almost all EEG parameters during the KDT. All dependent measures were affected by time.

## Discussion

Several lines of evidence implicate a role for distal temperature in declining vigilance in the evening [3-6]. The extent of foot cooling used in the current study was not sufficient to alter natural homeostatic thermal- and sleep-progressions, except for a transient and minor rise in wakefulness. More substantive cooling of the extremities might be required to impact vigilance; by affecting the regulated night-time reduction in T_C _or causing a distracting affect from T_sk _itself (which would aid wakefulness but may impair cognition).

## Conclusion

In healthy, young adults, T_C _and vigilance decline regardless of mild or moderate cooling of the feet, and any effect on sleepiness is small and transient.
